# Preparing Patients for Cosmetic Surgery and Aesthetic Procedures: Ensuring an Optimal Nutritional Status for Successful Results

**DOI:** 10.3390/nu15020352

**Published:** 2023-01-10

**Authors:** Tiziana Vitagliano, Pietro Garieri, Lidia Lascala, Yvelise Ferro, Patrizia Doldo, Roberta Pujia, Arturo Pujia, Tiziana Montalcini, Manfredi Greco, Elisa Mazza

**Affiliations:** 1 Department of Clinical and Experimental Medicine, University Magna Grecia, 88100 Catanzaro, Italy; 2 Department of Plastic Surgery, Hand Surgery and Reconstructive Microsurgery, Ospedale San Gerardo, 20900 Monza, Italy; 3 Department of Medical and Surgical Science, University Magna Grecia, 88100 Catanzaro, Italy; 4 Research Center for the Prevention and Treatment of Metabolic Diseases, University Magna Grecia, 88100 Catanzaro, Italy

**Keywords:** nutritional state, nutritional deficit, wound healing, cosmetic surgery, aesthetic procedures

## Abstract

Aesthetic and cosmetic medical practices have attracted considerable consumer attention globally. However, possible complications vary and range from mild, self-resolving ecchymoses or edema to more persistent complications. The aim of this review is to identify the nutritional deficits or excesses associated with the major complications of reconstructive surgery, aesthetic surgery, and mini-invasive aesthetic procedures. An additional goal is to provide a bundle of actions for professionals working in the industry in order to reduce the risks of aesthetic procedures and improve the clinical outcomes. Granulomas, hypertrophic scars and keloids, seromas, infections and xerosis, hyperpigmentation, petechiae, livedo reticularis, slower wound healing, and other poor outcomes are frequently associated with nutritional deficiencies. Nutritional status can markedly affect wound healing and tissue repair following surgical interventions, as well as the outcomes of aesthetic and cosmetic medical practices. Professionals working in this industry, therefore, need to consider the nutritional aspects of their patients to obtain the best results.

## 1. Introduction

Aesthetic medical practices are booming globally. With increased prevalence and the accessibility and evolution of cosmetic surgery in Western society, cosmetic plastic surgery has attracted a significant amount of attention from consumers. Given the influence that physical attractiveness has on our everyday lives, it is perhaps not surprising that many individuals seek a means to change or improve their appearance in order to conform to societal ideals of attractiveness. It has been suggested that individuals who have a strong self-image are more effective in work and social situations, and more comfortable in their relationships. This may at least partially explain the increasing recourse to aesthetic medical practices. Several studies have also shown that lower ratings of life satisfaction, increased media exposure to cosmetic surgery, time spent watching television, and religiosity are other significant predictors of the likelihood of undergoing cosmetic surgery [1].

Cosmetic surgery is a specialty within plastic surgery that focuses on appearance. Plastic surgery incorporates procedures that also restore form and function, such as breast reconstruction and burn surgery. Recently, non-surgical, minimally invasive options have grown in popularity, especially during the COVID-19 pandemic. In 2020, over 10 million procedures were performed, such as augmentation mammoplasty, facial implants and facial rejuvenation, blepharoplasty, facelifts, liposuction, abdominoplasty, lower body lifts, rhinoplasty, and Botox injections [2]. The top three minimally invasive cosmetic procedures performed were botulinum toxin injections, soft tissue fillers, and chemical peels [3].

Consumers have recognized the importance of valuing their overall health and wellness, which also includes aesthetic medicine. Furthermore, minimally invasive practices enable clinicians from a diversity of backgrounds to successfully enter the industry [4].

However, hematoma, seroma formation, nerve damage leading to sensory or motor loss, infection, scarring, blood loss and, in some cases, pulmonary embolism are the most frequent complications of aesthetic procedures which can also occur in any surgery [5]. Persistent granulomas, infections, and blindness due to inadvertent arterial injections are the most frequent complications with soft tissue fillers [5]. Analyzing the causes of adverse events following these procedures in the literature should help to make cosmetic procedures less risky.

Currently, nutrition plays a fundamental role in the surgical field, as in modern medicine, since an optimal nutritional status is now considered an important determinant of surgical outcome. A good nutritional status also optimizes healing and may lead to better results from all types of surgery [6]. Due to mineral and vitamin deficiencies, an inadequate nutritional status, in fact, can affect the three primary phases related to the healing processes after surgical procedures: inflammation, remodeling, and proliferative phase of the wound-healing process [7].

Previous research has shown that all forms of malnutrition are independent risk factors for surgical complications, including reduced wound healing, length of hospital stay, mortality, and costs [8,9].

Furthermore, a nutritional deficit can also lead to decreased immunity and inability to tolerate and overcome stressors, such as infections [10,11].

Nutritional status can markedly affect wound healing and tissue repair following surgical interventions [12].

Individuals who undergo cosmetic plastic surgery and aesthetic medicine span the nutritional spectrum from being patients who are generally healthy and nutritionally adequate to patients who are inherently catabolic, with chronic wounds, and nutritionally deficient [6]. It has been shown that up to 25% of plastic surgery outpatients are at risk of malnutrition [13]. Professionals working in this industry, therefore, need to consider the nutritional aspects of their patients in order to obtain the best surgical results.

The aim of this narrative review is to identify the nutritional deficits or excesses associated with the major complications of reconstructive surgery, aesthetic surgery, and mini-invasive aesthetic medical procedures. Another aim is to provide a checklist or bundle of actions for professionals working in this industry, so that they can reduce the risks associated with aesthetic procedures.

## 2. Major Complications of Reconstructive Surgery, Aesthetic Surgery, and Aesthetic Medicine 

Over 500 articles from 1982 to the present that are relevant to the topic were examined. Complications ranged from mild, self-resolving ecchymoses or edema to more persistent complications. Granulomatous occurred in 0.1–1% of patients undergoing filler injection procedures and appeared anywhere from 6 to 24 months after injection [14]. Approximately 10% to 20% of patients suffered a local complication following abdominoplasty [15]. In a series of over 1200 cases, the reported seroma rate was 15–31.2% [16,17]. Infection after breast implant surgery occurred in 1.1% to 2.5% of procedures performed for augmentation and up to 35% of procedures performed for breast reconstruction [18]. Infections were the second most common complication following abdominoplasty, with an estimated incidence between 1% and 3.8% [16], and the reported incidence of keloids and hypertrophic scars ranged between 1% and 3.7% [15].

### Link between Nutritional Status and Complications

*Granulomas*: Although adverse effects are rare, some patients may develop a foreign body reaction to such fillers. This usually manifests as a central region of macrophages surrounded by a zone of lymphocytes and a zone of fibroblasts. The presence of both epithelioid macrophages and multinucleated foreign-body giant cells with centrally arranged nuclei and surrounding collagen fibers tissue is a classic histological feature [19]. Inflammation is driven mainly by the innate immune system. This includes granuloma formation which is produced by several factors of the innate immune system as a response to a foreign body challenge, such as a filler. Malnutrition alters immune responses by decreasing T cell response [20], impairs phagocytic functions, and alters cytokine and antibody production [21,22]. Malnutrition is associated with granuloma development in patients affected by inflammatory bowel diseases [23]. Nutritional status could, thus, influence the appearance of granulomas.

*Hypertrophic scars and keloids*: These scar lesions are caused by chronic inflammation in the reticular dermis and are associated with excessive angiogenesis and abundant collagen accumulation. The risk factors associated with hypertrophic scar and keloid development, as well as aggravation, include female gender, estrogen exposure, hypertension, and familial history [24,25]. It is well recognized that unbalanced and incomplete diets affect skin health. Certain diets (such as those rich in hot and spicy foods) can aggravate surgery-induced inflammation [26]. Excessive alcohol consumption leads to enhanced radical formation in the human skin [27]. Once the concentration of free radicals in the body exceeds a critical level, cells or other skin components are damaged, especially the elastic fibers in elastin and collagen [28]. A low consumption of fruits and vegetables leads to a deficiency of certain micronutrients that are beneficial for the skin, which include vitamins or other molecules with antioxidant properties. Furthermore, the processing of plant foods also degrades the majority of vitamins and minerals [29]. It is possible that individuals with the conditions described above could develop some complications following aesthetic procedures.

*Seroma*: Despite the popularity of abdominoplasty surgery, patients are at a high risk of developing seroma, which is a complication of this type of surgery. It occurs in approximately 5% to 30% of patients and, in some cases, seroma may persist for months [30]. The pathophysiology of seroma formation concerns the extensive dissection in soft tissue and disruption of lymphatic and vascular ducts. The accumulation of fluids in the dead space between the flap of abdominal skin and the rectus muscle sheath often requires multiple aspirations through the skin. Body mass index (BMI) and a weakening of the abdominal wall (as in abdominal muscle loss) are the most important predicting factors of seroma formation after abdominoplasty [31]. Several surgical strategies have been proposed to reduce the rate of seroma formation. Certain conditions, such as advanced age, hypertension, high BMI, and low preoperative protein and albumin concentrations, predispose patients to develop a seroma [32]. Patients with either a low total protein serum level or a low albumin serum level tend to shift the plasma volume in both capillary and naturally empty cavities. Furthermore, in the case of low protein and albumin levels, the activation of macrophages through up-regulating immunoglobulin is altered [33]. A variety of lesions that complicate operations are the result of nutritional disturbances. Malnutrition is characterized by low levels of albumin [34], thus predisposing patients to skin complications.

*Infections*: Both obesity and underweight have been found to increase the infection risk in adults in a U-shaped manner [35]. Normal weight is, thus, associated with the lowest infection risk in most subjects. Skin infections are also the expression of an eating disorders (anorexia nervosa) [36]. Many physiological derangements may be present in patients with anorexia, and, therefore, a thorough history is needed during the preoperative assessment [37]. Following surgery, obese people are more susceptible to fatal complications. One study showed that one third of patients who experienced fatal complications after a surgery were obese, while nearly 15% were morbidly obese. Postoperative rates of wound infection were found to be 1.7 times higher in obese than non-obese patients, while four times higher rates of peripheral injury were reported in obese patients than in non-obese patients. It was suggested by the authors that obese patients who undergo outpatient procedure should be monitored in the hospital for several hours after surgery [38].

*Xerosis, hyperpigmentation, petechiae, livedo reticularis, slower wound healing, and other poor outcomes*: All these cutaneous manifestations could be the expression of the medical consequences of starvation, vomiting, or the abuse of drugs, such as laxatives and diuretics, which are often seen in psychiatric morbidity (eating disorders) [39]. Sarcopenia, which is defined as the loss of skeletal muscle mass and strength, is associated with frailty, death, and poor outcomes in both surgical and non-surgical patients [40,41,42]. Preventing malnutrition and promoting a healthy diet could be considered as basic surgical nutritional goals. Before surgery, these goals can be met through nutritional screening and assessment in order to diagnose, treat, and prevent malnutrition [43].

## 3. Key Nutrients for Individuals Undergoing Aesthetic Procedures

The nutritional status of individuals is influenced by their nutrient intake and use. The correlation between a healthy status and adherence to a good diet is now well established [44]. Early coordinated actions by surgical and dietary departments can provide optimal nutritional care to pre-surgical patients [45]. The same applies to subjects undergoing minimally invasive surgery and aesthetic techniques. Nutritional screening and assessments to diagnose, treat, and prevent all types of malnutrition should be carried out before surgery [45,46]. All essential nutrients should be, therefore, introduced to obtain the best results from aesthetic procedures.

*Protein*: Protein intake is necessary to introduce essential amino acids. These amino acids serve to prevent the use of muscles for energy purposes, resulting in protein malnutrition which has serious effects [47]. Amino acids are not only required for protein synthesis, but also play specialized roles in maintaining healthy skin [48]. An unbalanced amino acid ratio leads to a reduction in protein synthesis in the skin [48]. Essential amino acids are lost due to the shedding of stratum corneum cells. Protein intake, thus, prevents skin thinning, dehydration, and loss of skin elasticity, and reduces the appearance of sagging and wrinkles. During wound healing, adequate protein intake plays a key role in producing collagen. The consequences of protein depletion for wound healing include fibroblast proliferation and a decrease in angiogenesis [49]. This leads to a reduction in the synthesis and remodeling of collagen. Proteins are necessary for wound repair after a surgical procedure in order to maintain skin integrity, fluid and electrolyte balance, and activation of the immune response [50]. Specific amino acids, such as arginine and glutamine, have been shown to enhance wound healing. Arginine is a precursor to nitric oxide (NO) and proline, both of which are required for inflammatory response, collagen synthesis, and neovascularization [50]. For these reasons, arginine is one of the most recommended amino acids that accelerates the wound healing of injured skin [50] Glutamine also plays a role related to enzymatic, metabolic, antioxidant, and immune responses [51]. In wounds, glutamine leads to the up-regulation of some proteins that provide immunomodulatory and anti-inflammatory functions [52]. Glycine also plays an important role in promoting protein synthesis and wound healing, preventing tissue injury, and improving immunity [53]. Protein deficiency results in poor wound healing due to delayed progression from the inflammatory to the proliferative phase [54]. Protein deficiency leads to a delay in angiogenesis and a reduction in collagen formation, which decreases fibroblast activities [54]. In persistent wounds, there is an increase in protein requirements up to 250% as a result of the loss of a large number of proteins [55]. One study showed the importance of protein supplementation in reducing the incidence of complications following wound healing in patients with post-bariatric abdominoplasty [56]. Protein supplementation is, thus, important for improving the outcomes of plastic surgery.

*Omega 3 Fatty Acids*: Acting as a precursor for prostaglandins and facilitators of inflammation and metabolism, fatty acids are structurally essential in the lipid bilayer of cell membranes. Some studies have shown that Omega-3 polyunsaturated fatty acids (PUFA ω-3) facilitate wound healing [57,58]. PUFA ω-3 is composed of eicosapentaenoic acid (EPA), docosahexaenoic acid (DHA), and alpha-linolenic acid (ALA). The human body can obtain DHA and EPA only from fish that consume phytoplankton and marine algae that naturally produce them [57]. These fatty acids play a key role in anabolic processes and participate in the composition of the cell membrane, especially during regeneration following skin damage. It is possible that PUFA ω-3 modulates the local inflammatory response in wound areas, accelerating the rate of healing [59]. One study showed that diets enriched with PUFA ω-3 decreased the production of inflammatory mediators [58]. Furthermore, the quality of cutaneous wound healing in animals fed a diet enriched in PUFA ω-3 was compared to that of animals fed a standard diet. The animals fed the PUFA ω-3 enriched diet were found to have weaker wounds in the 30 days following an injury [59]. Data on the therapeutic effects of PUFA to promote wound healing and post-operative surgical recovery are very promising. PUFA ω-3 has positive effects on both depression and inflammation, supporting their potential importance in surgical patients.

*Micronutrients*: Several studies have investigated the potential value of specific micronutrients in regulating wound healing. The beneficial effect of adequate micronutrient intakes on surgical outcomes is also valid for minor plastic surgery. The correct intake of micronutrients is therefore essential.

*Vitamin A*: Vitamin A accelerates the renewal of the epidermis, affects the control functions of the skin, and exhibits a normalizing effect in keratinocyte differentiation [60]. Vitamin A increases collagen synthesis and cross-linking, the inflammatory response in wounds, and the quantity of immune cells in the wound environment to enable healing and skin cell differentiation [60]. Vitamin A also stimulates the transformation of low-activity fibroblasts in cells characterized by a fairly high production of collagen. The growth in the number and activity of fibroblasts has a positive effect on the state of the dermis, improving the elasticity, hydration, and firmness of the skin. Retinoids, such as retinol, improve skin elasticity, help in the removal of damaged elastin fibers, and promote angiogenesis. Patients affected by vitamin A deficiency have been found to be underestimated [60].

*Vitamin D*: Vitamin D is a fat-soluble secosteroid produced by the skin after exposure to sunlight. Dietary intake provides only 20% of the daily vitamin D requirement [60]. Vitamin D and its receptor are expressed everywhere in the body and has a number of effects on wound healing [61]. It is also necessary in the prevention of infection and consequent inflammatory response. Vitamin D enhances the immune system through the promotion of T helper type 2 cytokines, the down-regulation of cytokine generation, the reduction in interferon-mediated macrophage activation, the decreases in the production of T helper type 1 cytokines, and phagocytosis stimulation [62]. These overall effects improve inflammatory biomarkers and the level of oxidative stress [63]. Early identification in and treatment of patients who may be at risk of vitamin D deficiency is critical, especially in patients who have undergone bariatric surgery and who are referred for plastic procedures.

*Vitamin C*: Vitamin C is an essential cofactor for various enzymatic reactions and has strong antioxidant properties. During the hydroxylation of proline and lysine, vitamin C is important for collagen formation [60]. It also accelerates wound healing and contributes to bedsore healing. The combination of surgery procedures with pre-existing insufficient vitamin C status may lead to significant alterations in wound healing. Preclinical studies have shown that vitamin C supplementation results in higher expression of wound repair mediators and reduced expression of pro-inflammatory mediators for the early resolution of tissue remodeling and inflammation [64,65]. Vitamin C deficit can lead to capillary fragility, disturbances in the production of collagen, slower wound healing, and reduced resistance to infection, as well as scurvy [66].

*Vitamin E*: Due to its strong antioxidant properties, vitamin E is considered one of the main molecules that protect the body from oxidative stress. By preventing the oxidation of polyunsaturated fatty acids and promoting the synthesis of antithrombotic substances, vitamin E, in turn, prevents the development of atherosclerosis and microthrombi [60]. Vitamin E is an antioxidant molecule with anti-inflammatory properties as it inhibits phospholipase A2 activity, alters prostaglandin production, and results in reduced inflammation and collagen production. The exact role of vitamin E in wound healing is unknown and it has been linked to a delay in wound healing at high doses [60,67]. However, it has been suggested that vitamin E is involved in epithelization mechanisms, early inflammatory response and cell-mediated immunity, and angiogenesis [68]. Vitamin E deficiency can lead to premature skin aging and keratosis, as well as impaired wound healing [60].

*Other vitamins*: Vitamins B1 and B2 are both essential for an adequate production of collagen. As with other water-soluble vitamins, patients require a constant intake of these vitamins [66]. Vitamin K is important for normal clotting cascade. The production of prothrombin and factors II, VII, IX, and X are reduced if the level of vitamin K is low, and the initial phase of wound healing depends on blood clotting [69]. Vitamins B6 and B12 and folic acid degrade homocysteine, a toxic metabolic product, which promotes the healing of skin wounds. Their deficiency could, therefore, lead to a delay in healing from surgical procedures [70]. However, more research is needed to confirm the positive effects of the vitamin B group on wound healing.

*Zinc*: Zinc is an important nutrient in the DNA replication of cells, such as fibroblasts and epithelial cells. Other zinc functions in wound healing include the promotion of the immune response through lymphocyte activation and collagen production, fibroblast proliferation, and skin epithelization [71]. Zinc deficiency has been associated with poor wound healing and decreased wound strength in animal studies [72]. Agren et al. found that wound-breaking strength was significantly lower in the zinc-deficient group (-75% vs. controlled wounds) [72]. This is in accordance with the fact that zinc is an essential trace element in the early remodeling of scar tissue [72]. Serum zinc concentration is not representative of an individual’s zinc nutritional status.

*Iron*: Iron is a key cofactor in DNA replication and in the normal triple helix of collagen. Ferrous iron is a cofactor in the hydroxylation of lysine and proline in the synthesis of collagen [73]. There is a significant risk of iron deficiency in the population undergoing cosmetic surgery and aesthetic procedures. These individuals should, therefore, be screened for iron deficiency. However, it has been suggested that iron may delay wound healing through its free radical action [73]. The increase in free iron and reactive oxygen species released by neutrophils are important pathological steps, responsible for the increased destruction of connective tissue, persistent inflammation, and lipid peroxidation which contributes to the pro-oxidative environment of chronic wounds [73]. Iron may, thus, impair wound healing by contributing to toxic free radicals [49].

*Other minerals*: Important trace elements worth mentioning are copper and magnesium. Copper assists in the stimulation of angiogenesis through the promotion of vascular endothelial growth factor [74]. In an in vitro model, copper and zinc were shown to stimulate integrins from keratinocytes, which play an important role in wound healing [75]. It has been suggested that the mechanical properties of a scar can be enhanced by magnesium [76].

## 4. Dietary Pattern for Individuals Undergoing Aesthetic Procedures

The Mediterranean Diet (MD) and the Dietary Approaches to Stop Hypertension (DASH) are dietary patterns with a high nutritional quality; apart from their better anti-inflammatory effects and incorporation of high quantity of antioxidants, they also improve overall nutrition. The inclusion of foods typical of the MD/DASH is related to a better nutrient profile, with fewer individuals showing inadequate intakes of micronutrients [77,78]. These dietary patterns could, therefore, be used to prevent micronutrient deficiencies in the most vulnerable population groups. However, data from previous studies suggest a “theoretically” interesting association between a high adherence to MD/DASH and a low risk of aesthetic complications; unfortunately, to date, no studies have been carried out. Therefore, health promotion strategies focusing on promoting the MD/DASH, especially in population groups at risk of micronutrient deficiencies, still need to be developed.

## 5. Reduction in the Risk of Complications through the Bundle Approach

A bundle is a structured way of improving the processes of care and patient outcomes by creating awareness and helping to prevent complications. The theory is that enhancing teamwork and communication in multidisciplinary teams creates the necessary conditions for safe and reliable care (Table 1). Our screening questionnaire can be completed in less than one minute. The questions included in the screening tool have been validated in several studies [79,80,81], which help in drawing conclusions regarding the nutritional status and risk of complications. However, rather than validating the nutritional tool itself, we are interested in identifying the characteristics of patients at nutritional risk. Although many of the questions in the bundle are already known in the surgical field, based on the literature [3,5,6], they are associated with other questions on nutritional aspects. These questions investigate, in a fast and simple way, dietary patterns [29,44,60,82,83], weight loss [11,84,85,86], and some laboratory parameters [60,61,87] that suggest the presence of a nutritional deficiency and, therefore, an increased risk of complications from the procedures performed.

**Figure 1:**
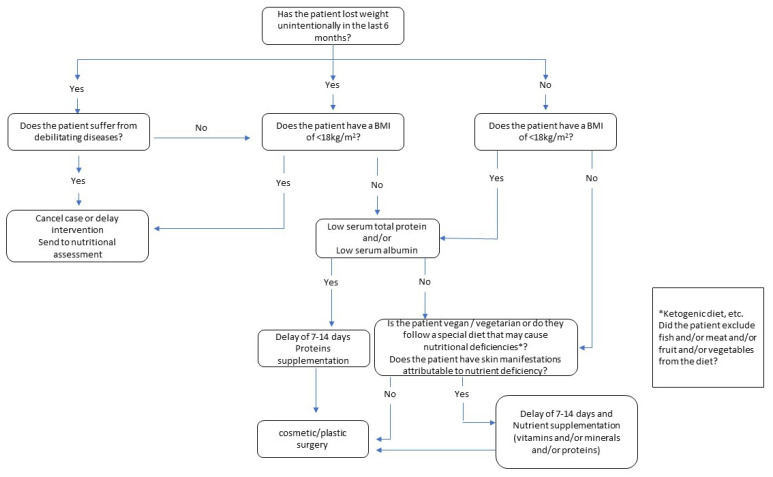
Decision flowchart for cosmetic surgery and aesthetic procedures.

We also propose a flowchart (Figure 1) that can be used as a guide through all the steps required in the selection of patients. This flowchart shows all the sequential steps and provides a systematic, visual display of the components of the decision process. Based on the literature on this theme, the flowchart should provide decision support for specialists in deciding whether to implement or postpone/cancel cosmetic or reconstructive surgery procedures.

In fact, there is a paucity of ‘gold standard’ evidence that nutritional support will reverse all the poor outcomes in this field. In some studies, a nutritional assessment is not performed and poor nutritional support is provided for short periods. However, there is good evidence that undernutrition and obesity, especially in surgical patients, are prospectively associated with an increased risk of poor outcomes after aesthetic procedures. Although our questionnaire is based on previous evidence, it still needs to be validated in a randomized controlled trial.

## 6. Conclusions

Only identifying individuals affected by obesity or malnutrition/sarcopenia is not enough but could contribute to reducing complications after plastic/cosmetic surgery or other aesthetic procedures. Patients with obesity/sarcopenia have worse surgical outcomes and pay a higher cost than patients with a normal nutritional status. Aesthetic medical professionals and surgeons in the sector should refer to a nutritionist to ensure their patient has an adequate dietary intake and to diagnose obesity or malnutrition before any procedures. We suggest the use of a preoperative bundle approach to prevent complications and that patients with remediable perioperative risk factors are enrolled in a training program, which will assist in improving outcomes, lowering costs, and reducing the length of stay.

## Figures and Tables

**Table 1:** Bundle of actions for professionals working in the industry in order to reduce the risks of aesthetic procedures and to improve the clinical outcomes.

− **Has the patient lost weight unintentionally in the last 6 months?** [84,85] − **Does the patient suffer from debilitating diseases?** [85,88] − **Does the patient have a BMI of <18?** [11] − **Does the patient have food intolerances/malabsorption?** [89,90] − **Does the patient have skin manifestations?** [91,92] − **Is the patient vegan/vegetarian or do they follow a special diet that may cause nutritional deficiencies?** [82] − **Does the patient have hypoalbuminemia/hypoproteinemia?** [87] − **Does the patient eat fruit and vegetables on a daily basis?** [29,60] − **Does the patient consume fish at least three times a week?** [44,83] − **Does the patient consume unprocessed/fresh foods daily?** [29] − **Is the patient taking vitamin/mineral and/or protein supplements?** [56,64,76] − **Does the patient get sufficient sunlight, but avoid the hottest hours?** [60,61]

BMI: Body Mass Index.

## Data Availability

Not applicable.
